# Interference with protease-activated receptor 1 does not reduce damage to subventricular zone cells of immature rodent brain following exposure to blood or blood plasma

**DOI:** 10.1186/s12952-014-0022-4

**Published:** 2015-02-04

**Authors:** Xiaoyan Mao, Marc R Del Bigio

**Affiliations:** Department of Pathology, University of Manitoba, and Children’s Hospital Research Institute of Manitoba, 401 Brodie Centre, 715 McDermot Avenue, Winnipeg, MB R3E 3P5 Canada

**Keywords:** Prematurity, Brain hemorrhage, Subventricular zone, Thrombin receptor, Coagulation factor II receptor, Cell proliferation

## Abstract

**Background:**

Prior work showed that whole blood, plasma, and serum injections are damaging to the neonatal rodent brain in a model of intracerebral/periventricular hemorrhage. Thrombin alone is also damaging. In adult animal models of hemorrhagic stroke, the protease-activated (thrombin) receptor PAR1 mediates some of the brain damage. We hypothesized that PAR1 interference will reduce the adverse effects of blood products on immature rodent brain and cells.

**Results:**

Cultured oligodendrocyte precursor cells from rats and mice were exposed to blood plasma with and without the PAR1 antagonists SCH-79797 or BMS-200261. In concentrations previously shown to have activity on brain cells, neither drug showed evidence of protection against the toxicity of blood plasma. Newborn mice (wild type, heterozygous, and PAR1 knockout) were subjected to intracerebral injection of autologous whole blood into the periventricular region of the frontal lobe. Cell proliferation, measured by Ki67 immunoreactivity in the subventricular zone, was suppressed at 1 and 2 days, and was not normalized in the knockout mice. Cell apoptosis, measured by activated caspase 3 immunoreactivity, was not apparent in the subventricular zone. Increased apoptosis in periventricular striatal cells was not normalized in the knockout mice.

**Conclusion:**

Interference with the thrombin-PAR1 system does not reduce the adverse effects of blood on germinal cells of the immature rodent brain. PAR1 interference is unlikely to be a useful treatment for reducing the brain damage that accompanies periventricular (germinal matrix) hemorrhage, a common complication of premature birth.

## Background

Hemorrhage in the periventricular germinal tissue of developing brain (often called germinal matrix or periventricular hemorrhage, PVH) is a major complication of preterm birth before 32 weeks gestational age [1]. PVH is associated with suppressed proliferation of the periventricular germinal cell populations in human infants [2]. The same suppression occurs in an experimental mouse model [3]. It is important to understand this phenomenon because it may adversely affect subsequent brain development and contribute to the neurological complications suffered by premature infants [4]. Blood injections have been shown to damage immature mouse brain; much of the effect seems to be attributable to the plasma proteins thrombin and plasmin [5,6]. Using cultured rat subventricular zone (SVZ) cells and oligodendrocyte precursor cells (OPC) we showed that blood plasma and blood serum, as well as purified thrombin, plasmin, and kallikrein had similar toxic effects on cell proliferation, migration, and differentiation [7].

Prothrombin is a serine protease contained in blood plasma. Following activation, thrombin has a central in the blood coagulation cascade. It also promotes inflammation and acts as a mitogen for some cell types [8]. In adult animal models of brain hemorrhage, thrombin plays a role in the resulting brain damage [9]. Signaling through one of the major G-protein coupled receptors, protease activated receptor 1 (PAR1; properly called coagulation factor II receptor, F2r), appears to mediate the process [10,11]. Interference with this pathway has been proposed as a potential target for therapeutic intervention following brain hemorrhage. Following blood injection into 1-day-old mouse brains, the thrombin inhibitor hirudin was capable of reducing inflammation and brain cell death at 2 days, but the long-term outcomes were unchanged [6]. In the rat brain cell model system, hirudin reduced the cell death caused by thrombin but not the suppression of cell proliferation [7]. Plasmin, which was shown to be damaging in the above-mentioned mouse and cell culture models, can also act through PAR1 [12].

Cultured OPC express high levels of PAR1 messenger RNA with expression declining as the cells mature, and they show PAR1 and PAR2 immunoreactivity at the O4+ stage of maturation [13]. Cultured OLN-93 oligodendrocyte cells also express PAR1 and PAR3, but not PAR2 or PAR4 [14]. Thrombin stimulation causes increases in intracellular calcium ion in SVZ-derived OPC and the effect is mediated by PAR-1 activation [15]. SCH-79797 suppresses PAR1 signaling in primary astrocyte cultures [16,17] and in hippocampal slice cultures [18] and it protects against brain injury in rats [19]. BMS-200261 interferes with PAR1 in cultured astrocytes [12] and reduced infarct volume in a mouse model of focal cerebral ischemia [20]. We hypothesized that interference with the PAR1 will reduce the germinal cell damage associated with blood injection into the immature rodent brain. The first aim was to determine if chemical PAR1 antagonists could reduce damage to cultured mouse and rat OPC exposed to blood plasma. The second aim was to compare periventricular SVZ damage following blood injection into brains of newborn wild type, heterozygote, and PAR1 knockout mice. In mice this region rapidly involutes between birth and 8 days age [21]. Cells born here are destined to become oligodendrocytes and astrocytes in the cerebrum and neurons in the olfactory bulb [22-25].

## Results

### OPC cultures exposed to blood plasma and PAR1 inhibitors

**Figure 1:**
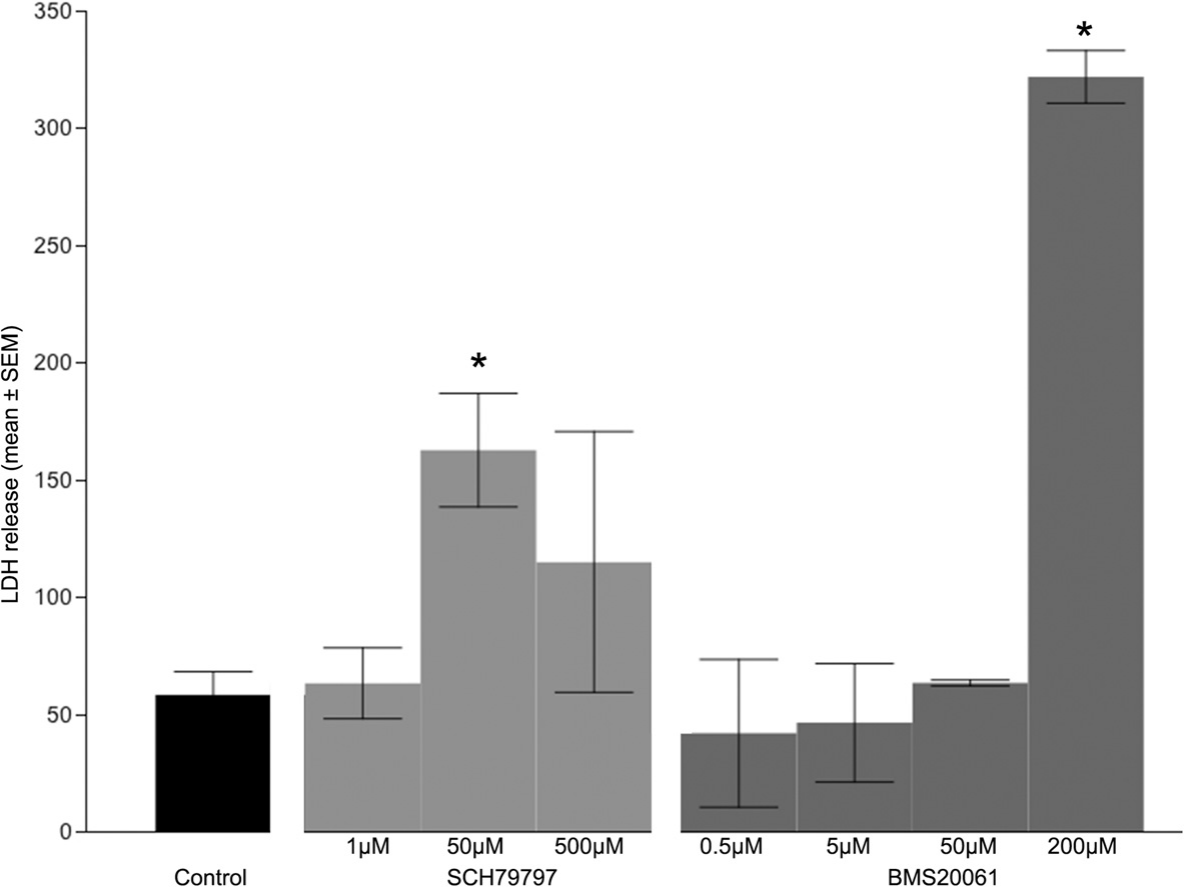
**Bar graph showing effects of PAR1 inhibitors on rat oligodendrocyte precursor cells (OPC) cultured in serum-free defined media.** Cell death was measured by LDH release (optical density units; mean ± SEM). At high concentrations both SCH79797 and BMS20061 were toxic to the cells (*p < 0.05).

**Figure 2:**
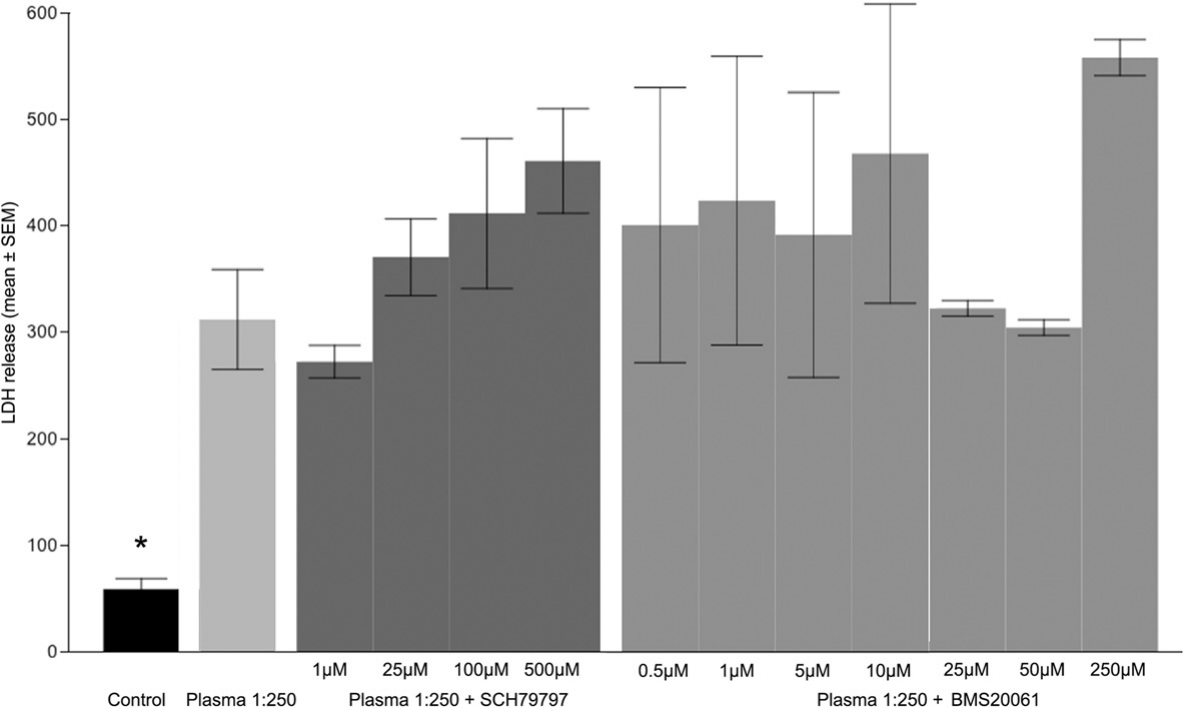
**Bar graph showing effects of plasma alone and in combination with PAR1 inhibitors on rat oligodendrocyte precursor cells (OPC) in culture.** Cell death was measured by LDH release (optical density units; mean ± SEM). Plasma at 1/250 concentration added to the defined culture media was toxic to OPC as indicated by increased LDH release. There was no evidence for protection with SCH79797 and BMS20061 at any dose. Control values (no plasma) were significantly less than all groups (*p < 0.05) and there were no statistically significant differences between treatment groups (all comparisons p > 0.5).

Both the rat and mouse OPC cultures expressed predominantly oligodendrocyte lineage markers, including A2B5 at 6 hours, O4 for up to 3 days, and CNPase and MBP after 6 days. In the absence of plasma, exposure of rat OPC to SCH-79797 at concentrations of 0.05 to 5 μM had no obvious affect on LDH release, while 10-50 μM was associated with a 2-fold increase in LDH release (P < 0.05). In the absence of plasma, exposure of rat OPC to BMS-200261 at 0.05 to 50 μM had no obvious affect on LDH release, 100 μM was associated with a 2-fold increase in LDH release, and 200 μM was associated with a 4-fold increase in LDH release (p < 0.05; Figure 1). Exposure of rat OPC to plasma at 1:1000 concentration was associated with a 2-fold increase in LDH release, 1:250 was associated with a 3.5-fold increase in LDH release, and 1:100 was associated with a 6-fold increase in LDH release. Combined with plasma at 1:1000, 1:250, or 1:100 concentrations, SCH-79797 showed no evidence of protection at 0.05-5 μM concentrations. Combined with plasma at 1:250 concentration, 10-50 μM SCH-79797 accentuated the toxicity (4-fold increase in LDH release). Combined with plasma at 1:250 concentration, BMS-200261 showed no evidence of protection at 0.05-10 μM while 25-200 μM accentuated the toxicity (4-fold increase in LDH release) (Figure 2). Cell proliferation measured by BrdU incorporation was not normalized with the drug therapy (all comparisons p > 0.5).

The mouse OPC experiment was abbreviated with avoidance of the high concentrations of drug that were independently toxic to the rat OPC. Exposure to blood plasma at 1:250 concentration was associated with a 5-fold increase in LDH release from mouse OPC. Exposure of mouse OPC to SCH-79797 at 5-10 μM caused a slight increase in LDH release; combined with plasma at 1:250 the toxic effect was doubled (10-fold increase LDH release). Exposure of mouse OPC to plasma at 1:250 concentration combined with BMS-200261 at 0.5 and 2 μM reduced the LDH release in comparison to plasma alone, however the difference was not statistically significant (194 ± 88 vs. 51 ± 18 U; p = 0.22).

### PAR1^−/−^ mice with autologous blood injection into brain

Blood injected mice suffered no mortality and there was no obvious behavioral consequence. In intact and sham injected control mice (wild type, heterozygote, and PAR1^−/−^), Ki67 was expressed by 23.5 ± 4.8% of SVZ cells along the frontal horns of the lateral ventricles. There were no significant differences between the controls at the two time points or between genotype. Only scattered cells outside of the SVZ were Ki67 positive. In the controls, activated caspase 3 immunoreactivity was negligible. Microglia with delicate processes that expressed Iba1 were scattered throughout the cerebrum. However, within the SVZ only a small proportion (1.4 ± 0.2%) of cells expressed Iba1. There were no statistically significant differences across genotype or time (all p > 0.4).

**Figure 3:**
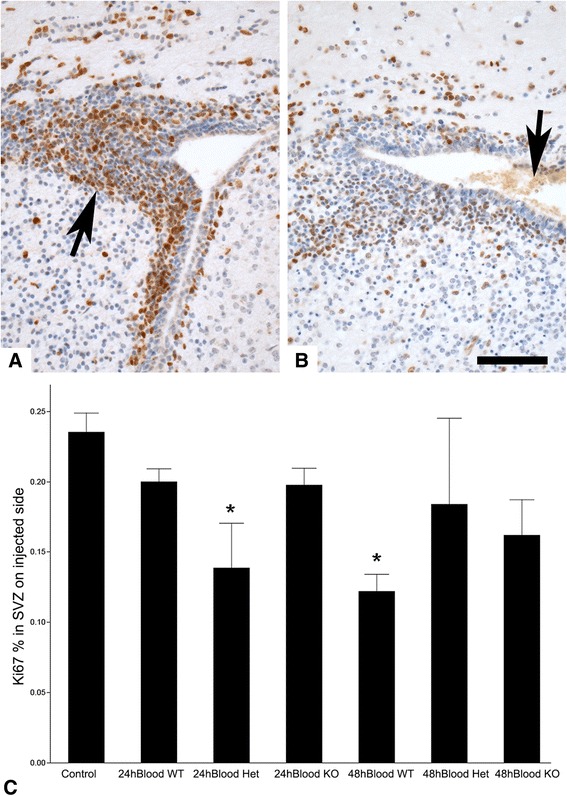
**Cell proliferation in immature mouse brain following periventricular blood injection. A** - Ki67 nuclear immunoreactivity (brown stained nuclei; hematoxylin counterstain) is prominent in the frontal periventricular subventricular zone (SVZ, arrow) of control (i.e. no blood injection) mice from all three genotypes (this example is wild type). **B** – Following blood injection, some of which extends into the frontal horn of the lateral ventricle (arrow), Ki67 immunoreactivity in the SVZ is reduced (this example is a PAR1 knockout). **C** – Quantitative analysis (ANOVA) shows the proportion of Ki67 immunoreactive cells in the SVZ was significantly reduced in the 24-hour heterozygous (Het; *p = 0.0050) and the 48-hour wild type (WT; *p = 0.0054) mice; the reduction also approached significance in the 48-hour knockout (KO; p = 0.0577) in comparison to controls. Bar = 100 μm.

Following blood injection into the right periventricular region, blood entered the frontal horns of the lateral ventricles, which enlarged slightly. Ki67 expression by proliferating cells decreased in the ipsilateral SVZ. In comparison to controls, the differences were statistically significant for the 24-hour heterozygous and the 48-hour wild type mice (p < 0.005; Figure 3A-C). Following blood injection, suppressed Ki67 expression in the PAR1^−/−^ mice with did not differ from the wild type or heterozygous mice at either time point (p = 0.40 to 0.95). In the contralateral SVZ the only significant difference in Ki67 expression after blood injection was a slightly lower percentage in the PAR1^−/−^ mice at 24 hours (19.3 ± 2.5% vs. 28.4 ± 5.8% in heterozygote control, p = 0.0418; 26.6 ± 3.1% in wild type control, p = 0.136); at 48 hours there were no differences between groups (p > 0.7).

**Figure 4:**
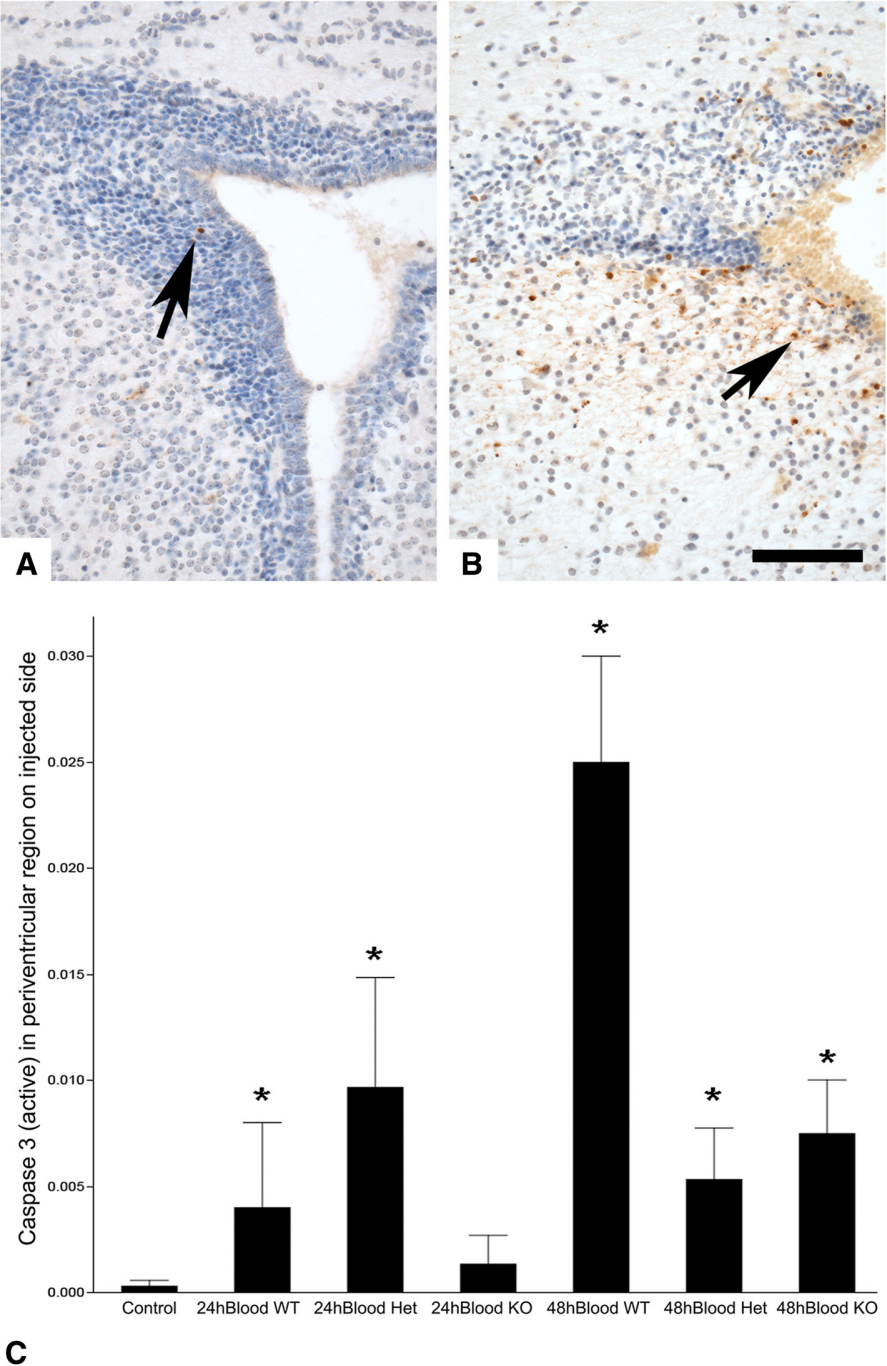
**Cell death in immature mouse brain following periventricular blood injection.** **A** - Activated caspase 3 immunoreactivity is extremely rare in the SVZ of control mice (brown stained cells; hematoxylin counterstain) (arrow; this example is wild type). **B** – Following blood injection, activated caspase 3 immunoreactivity is prominent in the striatum (arrow) but very rare in the SVZ (this example is a PAR1 knockout). **C** – Quantitative analysis shows a statistically significant increase in caspase 3 immunoreactive cells in the striatum of most groups in comparison to intact control (p < 0.007), but there were no significant differences between the blood-injected groups (all p > 0.15; Wilcoxon method). All micrographs were taken at 400× magnification. Bar = 100 μm.

In comparison to mice without blood injection, activated caspase 3 immunoreactive cells were significantly increased in the heterozygous mice at 24 hours (and the wild type mice at 48 hours after blood injection (Figure 4A-C). Careful evaluation of the H&E stained sections demonstrated only very rare karyorrhectic nuclei. Most (>80%) immunoreactive cells were in the striatum and very few were in the SVZ. This is similar to our previous observation in mice that blood injection substantially suppresses SVZ proliferation but has only minor effects on cell death [3]. The PAR1^−/−^ mice with blood injection did not differ from controls that had not received blood injection, and they had significantly fewer caspase 3 positive cells in the periventricular striatum (but not SVZ) at 48 hours in comparison to blood-injected wild type mice (p = 0.0028). The general trends were similar for activated caspase 3 positive in the cerebral white matter, but none of the differences were statistically significant (all p > 0.2).

Following blood injection, the quantity of Iba1 expressing cells in the SVZ was significantly lower in most groups in comparison to intact baseline (e.g. 0.6 ± 0.2% in 48 h blood-injected heterozygote vs. 1.4 ± 0.2% in intact control; p = 0.0326). However, there were no differences between genotypes following blood injections (all comparisons >0.13) and considering the very low baseline values it is not clear that the decrease after blood injection is biologically important. There was no numeric change in the cerebral white matter following blood injection in any group. However, the immunoreactive cells tended to be larger suggesting that the microglia had reacted to the needle insertion and/or blood injection.

## Discussion

Blood, blood plasma, blood serum, plasmin, and thrombin were previously shown in our laboratory to suppress proliferation and cause cell death in rat OPC in a dose-dependent manner [7]. Neonatal mouse brain showed comparable sensitivity in vivo following injection of blood components including suppressed cell proliferation in the SVZ [3,5,6]. PAR1 has repeatedly been shown to play an important role in hemorrhagic brain damage in adult experimental models [9,10,26,27]. However, based on the experiments presented here, we reject the hypothesis that PAR1-mediated signaling is critical to the SVZ changes that follow brain hemorrhage. Blood plasma was applied to OPC at a concentration previously shown to cause a combination of reduced cell proliferation and increased cell death [7]. At concentrations previously shown to have activity in astrocytes [12,17] and hippocampal slices [18], the selective peptide (BMS-200261) and nonpeptide (SCH-79797) inhibitors of the PAR1 receptor failed to protect mouse or rat OPCs against the toxic effect of blood plasma, despite the fact that these cells are known to express PAR1 and respond to its activation [13,15]. The chemicals used have both been shown to suppress PAR1 signaling in cultured brain cells [12,16,17] and to protect against brain injury in adult rodents [19,20]. At high concentrations the PAR1 inhibitors themselves were toxic. SCH-79797 (200 nM) has been shown to cause apoptosis in 3 T3 fibroblast cell cultures through PAR1 independent mechanisms [28]. One likely reason why the PAR1 inhibitors were not protective is the fact that blood plasma contains many other injurious constituents that act through non-PAR1 mediated mechanisms. We have previously shown in this OPC culture model, that cells are also suppressed and killed by complement 5a, gamma amino butyric acid, and kallikrein [7].

In the neonatal mouse experiment, injection of autologous blood into the periventricular region of the brain caused a substantial suppression of proliferating SVZ cells as well as a slight increase in apoptosis among non-SVZ periventricular cell populations. This is similar to the findings in our previous experiments using this model system [3,5]. In the current experiment, the damaging effect of blood on the periventricular SVZ was not modified through selective omission of the thrombin receptor PAR1. However, there was a decrease in caspase 3 activation (presumably an indicator of apoptosis) within the striatum, where the cells are more mature. Adult PAR1^−/−^ mice have less brain damage following intracerebral injection of thrombin [10], transient occlusion of the middle cerebral artery [20,29], global ischemia [30], and following cortical stab injury [26]. The absence of a protective effect on SVZ cells in vivo might also relate to the distribution of PAR1 in the developing brain. During mouse development, PAR1 plays an important role in vasculogenesis and hemostasis [31], as well as more focal involvement in neural tube closure [32]. Reports on expression of PAR1 mRNA in developing rodent brain are not entirely consistent. In one mouse brain database, PAR1 is expressed mainly on endothelial cells and a subpopulation of SVZ cells on gestational day 15, while on postnatal day 7 most PAR1 expression is in neurons [33]. In another mouse database, ganglionic eminence progenitors are reported to have high expression on gestational day 16 [34]. In the rat, PAR1 mRNA is abundant in the periventricular region of brain at gestational day 14 and the day of birth, while discrete neuronal expression is predominant by postnatal day 7 [35].

Shortcomings must be considered. Despite the value of mouse experiments for genetic manipulation, newborn mouse is not the ideal animal model for studying germinal matrix damage following PVH. In humans, premature birth at 24–28 weeks gestation is the period of greatest risk for PVH [1]. In some respects the postnatal day 6–7 mouse brain is comparable to human brain at that stage of development [36]. However, in terms of relative volume, the ganglionic eminence (the largest SVZ concentration) of the gestational day 17 mouse and gestational week 24 human are more comparable [2,21]. Furthermore, the mouse SVZ is much less complex than in primates [37,38]. The proportion of proliferating cells in the human ganglionic eminence SVZ at 24 weeks gestation is approximately 60% [2]. In neonatal mice the proportion is much lower at ~23%. We focussed on PAR1 because most experimental evidence shows a role for that receptor in brain damage related to hemorrhage. However, PAR2 [39], PAR3 [40], and PAR4 [41] also appear to play roles in microglial activation and neuron degeneration that follow experimental brain injury. Small sample sizes must be considered. In the OPC culture experiments, toxicity of the PAR1 inhibitors was not strictly dose dependent suggesting some unexplained variation in the system. However, the inhibitors showed no trends suggesting protection. Despite small sample sizes in the mouse experiment the basic cellular responses to blood injection (reduced SVZ proliferation, increased cell death in the adjacent striatum) were in the predicted directions and there were no trends suggesting that the PAR1 knockouts had any reduced damage in the SVZ. Failing to identify any positive effects in two kinds of experiments, analysis of PAR1 role in these model systems was not exhaustive, however rejection of the hypothesis is reasonable.

## Conclusions

We conclude that blocking the thrombin-PAR1 system does not reduce the adverse effect of blood and blood plasma on SVZ cells and OPC of the developing rodent brain, despite the published evidence for expression and functionality of PAR1 in these cell populations. These experiments might not exclude an important role of thrombin, but they do show that thrombin-induced damage through PAR1 is not the exclusive mode of injury following exposure to blood plasma, which contains many active and potentially toxic proteins and catabolic products [42]. Despite published evidence in adult model systems, protection of immature brain against the adverse effects of hemorrhage by interference of a single molecular pathway should be considered unlikely.

## Methods

### Reagents

Dulbecco’s Modified Eagle’s Medium (DMEM), poly-D-lysine (PDL), putrescine, thyroxin, tri-iodothyroxine, progesterone, sodium selenite, papain, deoxyribonuclease l, bovine serum albumin (BSA) fraction V, and insulin were purchased from Sigma-Aldrich. L-glutamine, 0.25% trypsin-EDTA, and fetal bovine serum (FBS) were purchased from Invitrogen/Life Technologies. MEM was purchased from Gibco/Life Technologies. Holo-transferrin (human) was purchased from EMD Millipore. Trypsin inhibitor was purchased from Roche Life Science. FGF-basic and EGF were purchased from Peprotech. PDGF-AA was purchased from R&D Systems Inc.

### Animals

All experiments were approved by the University of Manitoba Animal Care Committee. Sprague–Dawley rats and wild type C57BL/6 mice were bred at the Central Animal Care facility of the University of Manitoba. Heterozygous PAR1 knockout mice (B6.129S4-*F2r*^*tm1Ajc*^/J) on a C57Bl/6 background were purchased from Jackson Laboratories (Stock Number: 002862). Animals were bred to obtain homozygous PAR1 knockout mice. The genotype of newborn mice was determined from tail samples. Genomic DNA was extracted using proteinase K in a DNA digestion buffer. Polymerase chain reaction (PCR) was used to detect PAR1 using the specific primers 5′GAC GTT CAG AGG AAG GCT GA3′ (oIMR3504, Wild type), 5′AAA ATG AAA GCG TCC TGC TG3′ (oIMR3503, Common) and 5′TGG ATG TGG AAT GTG TGC GAG3′ (oIMR8162, Mutant). PCR products were separated by electrophoresis in a 2% agarose gel and stained with Gelstar Nucleic Gel stain. The expected bands are wild type = 175 base pairs (bp), mutant = 225 bp, and heterozygote = 175 bp / 225 bp.

### Cultures of rat and mouse oligodendrocyte precursor cells (OPC)

Rat OPC were cultured as previously described in detail [7]. Mouse OPC cultures required some modifications [43]. Wild-type mice were decapitated at 1-day age, the brain was removed and stripped of meninges, the cerebral hemispheres (excluding hippocampus and basal nuclei) were isolated, and cells were dissociated in DMEM medium supplemented with 5000U penicillin and streptomycin and Accutase cell detachment solution (Millipore). Cells were washed and resuspended in DMEM culture medium supplemented with 4 mM L-glutamine, 5000U penicillin and streptomycin, 20% heat inactivated fetal bovine serum (FBS), 10 ng/ml basic fibroblast growth factor (bFGF), 10 ng/ml epidermal growth factor (EGF) and 5 ng/ml of platelet-derived growth factor (PDGF-AA) then grown in lysine-coated flasks. On day 7–8, DMEM medium with L-glutamine, penicillin and streptomycin, 20% FBS, and 10 ng/ml PDGF was used. Cultures were kept at 37°C in humidified chamber with 5% CO_2_; the media was changed every 3–4 days. OPCs were isolated from the mixed glial culture by shaking on day 14–18. Microglia were removed by allowing them to attach in a plastic Petri dish. Floating OPCs were cultured on glass coverslips in Sato’s serum-free modified medium, which contained 20 ng/ml PDGF-AA, putrescine (Sigma P5780) 0.16 μg/ml, thyroxin (T4, Sigma T-1775) 4 ng/ml, tri-iodothyroxine (T3, Sigma T-6397) 4 ng/ml, progesterone (Sigma P8783) 0.66 ng/ml, bovine serum albumin (BSA) fraction V (Sigma A4919) 1 μg/ml, L-glutamine (GIBCO), human holotransferrin (Sigma T0665), Penstrep (Invitrogen 15140), and insulin (Sigma I1882). In pilot experiments (results not shown), oligodendrocyte lineage was verified after 6 hours, 3 days, and 6 days by fixing cells in cold methanol and 4% paraformaldehyde, followed by immunofluorescence using primary antibodies to A2B5 (mouse IgM; Millipore MAB312R), myelin basic protein (MBP, rabbit IgG; Santa Cruz SC-13914), 2′3′-cyclic-nucleotide 3′-phosphodiesterase (CNPase, mouse IgG; Millipore MAB326), O4 (mouse IgM; Neuromics MO15002), and glial fibrillary acidic protein (to exclude astrocytes, rabbit IgG; Novocastra Z0334) and appropriate secondary antibodies with 4′,6-diamidino-2-phenylindole (DAPI; Sigma, D-8417) labeling of nuclei.

### Cultured oligodendrocyte precursor cell (OPC) cell death assays

We previously showed that plasma at 1:100 dilution had a negative effect on OPC proliferation and caused cell death detectable by both lactate dehydrogenase (LDH) release and activation of caspase 3. Purified thrombin at the calculated equivalent dose had a slightly greater (although not statistically significant) adverse effect [7]. Adult mouse and rat blood were collected in EDTA-coated tubes (to prevent blood clotting) and plasma was isolated as previously described [7], then aliquoted and stored at −20°C. When the thawed plasma is added to Ca^++^-containing cell culture medium plasma enzyme activity is normalized [44], although fibrinogen activity might not be entirely restored [45]. Isolated OPCs from rat and wild type C57Bl6 mice in serum-free Sato’s culture medium were plated in 24-well plates with PDL-coated glass coverslips (1×10^5^ or 2×10^5^ cells/well in different trials) and allowed to adhere. Four hours later the media was changed to serum-free Sato’s medium with added 10 μM bromodeoxyuridine (BrdU Sigma B-5002) with or without added PAR1 antagonists SCH-79797 dihydrochloride (Tocris, Ellisville, MO, USA) or BMS-200261 (Sigma). The dose range was based on previously published work. SCH-79797 modulated the response of cultured astrocytes to plasmin at a concentration of 5 μM [17] and thrombin-mediated cell death in hippocampal slices at 10 μM [18]. BMS-200261 blocked the plasmin-induced rise of cytoplasmic Ca2+ in cultured astrocytes at 1 μM [12], The cells were simultaneously exposed to rat or mouse blood plasma (1:100, 1:250, and 1:1000 dilutions) for 24 h. Incorporated BrdU was detected with monoclonal mouse anti-BrdU antibody (Cell Signaling# 5292) and cells were counterstained with DAPI. Cells were counted with a fluorescence microscope using a 20x objective magnification. Prior to fixation, 5 μl supernatant from OPC cultures was transferred to a 96-well plate, and 50 μl of the LDH cytotoxicity detection kit II (Biovision) reaction mixture was added. After 30 minutes absorbance was recorded at 450 nm using microplate reader. LDH concentration was calculated in triplicate according to standards with background subtraction. All rat OPC tests were repeated in triplicate from separate cell isolations and all results were read at 30 minutes and 3 hours. All mouse OPC tests were done in duplicate. Data, expressed as mean ± standard error of mean, were compared using ANOVA.

### Autologous blood injection into neonatal mouse cerebrum

PAR1 mutant heterozygote mice mating typically yielded 2–3 neonates of each genotype (PAR1^−/−^, PAR1^+/−^, PAR1^+/+^) per litter. Mice were housed with their mothers. As previously described [3,6], 1-day-old mice were anesthetized with 2% isoflurane, whole blood was obtained from the tail and then was injected (15 μl though a 30 gauge needle) into the right periventricular region/striatum. The injection coordinates were 1.5 mm lateral to midline, 0.5 mm posterior to the outer canthus of the eye, and 2.5 mm depth. Syringes contained no anticoagulants and procedures were completed within 2 minutes before the blood clotted. We previously showed that serine protease activity due to thrombin and/or plasmin is elevated at the edge of the resulting hematoma [6]. Additional controls included sham injected and intact brain of all genotypes. Mice were killed by isoflurane overdose followed by exsanguination and transcardiac perfusion with cold 4% paraformaldehyde in phosphate buffered saline 24 or 48 hours after blood injection. The brains were removed, left overnight in the same fixative, sliced in the coronal plane encompassing the blood injection site, dehydrated, and embedded in paraffin. Brains were assessed at 24 and 48 hours after blood injection (3 genotypes × 2 time points × 2 conditions; 3–4 per group).

Brain slices were sectioned serially at 5 μm thickness and every 10th slide was stained with hematoxylin and eosin (H&E) to identify the center of the injection site. In previous similar experiments we determined in advance several exclusion criteria to be determined by microscopic examination: 1) sham injections with blood occupying the striatum or ventricle; 2) blood injections with no obvious blood collections or hematoma not involving the SVZ. In this experiment no exclusions were mandated. Deparaffinized sections were treated using heat-mediated antigen retrieval (0.1 M citrate buffer, pH6, for 20 minutes) and endogenous peroxidase was suppressed with 3% H_2_O_2_ in methanol for 10 minutes. Sections were blocked with 10% goat serum in PBS. Immunohistochemical staining was used to detect reactive astrocytes (rabbit anti-GFAP, 1:3000; Novocastra Z0334), proliferating cells (rabbit anti-Ki67, 1:2000; Novocastra Labs NCL 301103), and apoptotic cells (rabbit anti-activated caspase-3, 1:250; Gene Tex GTX22302). Microglia were detected with rabbit anti-Iba1 (1:2000; Wako, Osaka, Japan 019–19741), and reactive changes were evaluated by cell hypertrophy and increased cell density in comparison to the contralateral side. Immunoreactive cells were revealed using biotinylated goat anti-rabbit secondary (Jackson IR, 111-065-144) followed by streptavidin/horseradish peroxidase (HRP; Jackson IR 016-030-084), and diaminobenzidine (DAB with 10 μL 30% H_2_O_2_). After hematoxylin counterstain, sections were dehydrated and coverslipped.

The evaluator was blinded to the genotype. Immunoreactive cells and total cells were counted at 400x magnification in two anatomical regions bilaterally (ipsi- and contralateral to the injection site) using an ocular counting grid. The subventricular zone (SVZ) between the frontal horn of the lateral ventricle and the striatum and at the angle of the frontal horn was evaluated in a 50×200 μm rectangle and an approximately 200×200 μm triangular area. Dorsal white matter above the ventricle was evaluated in a 150×500 μm rectangular area. The number of cells immunoreactive for each antigen was calculated and the means were compared. For the Ki67, which had a normal distribution, we used ANOVA followed by Dunnett’s method for comparison to controls without blood injection and Tukey-Kramer tests for comparison to matched groups with blood injection. The caspase 3 and Iba1 values did not have normal distributions, therefore we used the nonparametric Wilcoxon method to compare groups (JMP10 software; SAS Institute, Cary NC). Differences were considered significantly different at p < 0.05.

## Abbreviations

ANOVA: analysis of variance
CNPase: 2′,3′-cyclic-nucleotide 3′-phosphodiesterase
DMEM: Dulbecco’s Modified Eagle’s Medium
LDH: Lactate dehydrogenase
MBP: Myelin basic protein
OPC: Oligodendrocyte precursor cell
PAR1: Protease activated receptor 1
PVH: Periventricular hemorrhage
SVZ: Subventricular zone
